# Genetic characterization of native and introduced populations of the neotropical cichlid genus *Cichla* in Brazil

**DOI:** 10.1590/S1415-47572009005000060

**Published:** 2009-09-01

**Authors:** Daniel Cardoso de Carvalho, Denise Aparecida Andrade de Oliveira, José Enemir dos Santos, Peter Teske, Luciano B. Beheregaray, Horacio Schneider, Iracilda Sampaio

**Affiliations:** Departamento de Zootecnia, Escola de Veterinária, Universidade Federal de Minas Gerais, Belo Horizonte, MGBrazil; 2 Programa de Pós-Graduação em Zoologia de Vertebrados, Pontifícia Universidade Católica de Minas Gerais, Belo Horizonte, MGBrazil; 3 Molecular Ecology Lab, Department of Biological Sciences, Macquarie University, SydneyAustralia; 4 Instituto de Estudos Costeiros, Universidade Federal do Pará, Bragança, PABrazil; 5 School of Biological Sciences, Flinders University, AdelaideAustralia

**Keywords:** peacock bass or tucunaré, mtDNA, invasive populations, Amazon basin, Minas Gerais

## Abstract

A molecular phylogenetic analysis based on mitochondrial 16S ribosomal DNA and Control Region sequences from native and introduced populations was undertaken, in order to characterize the introduction of *Cichla* (peacock bass or tucunaré) species in Brazil. Mitochondrial DNA haplotypes found in introduced fish from Minas Gerais state (southeastern Brazil) clustered only with those from native species of the Tocantins River (*Cichla piquiti* and *C*. *kelberi*), thereby suggesting a single or, at most, few translocation acts in this area, even though with fish from the same source-population. Our study contributes to an understanding of the introduction of *Cichla* in regions of Brazil outside the Amazon basin, and adds phylogenetic data to the recently describe *Cichla* species, endemic from the Tocantins-Araguaia basin.

## Introduction

The introduction of non-native species is considered to be the second greatest threat to native biodiversity, after habitat loss (UNEP 2005). Pimentel *et al.* (2005) estimate that non-native or indigenous species represent a cost of approximately $120 billion per year in damages and control for the USA alone. In addition to the economic impact, invasive species imply severe negative consequences for native fish species in southern Brazil. One particularly important example of this is the Amazonian cichlid genus *Cichla*, the species of which are collectively known in Brazil as tucunaré. Introduced tucunaré appear to have been responsible for the local extinction of the characoid fish species *Metynnis* cf. *roosevelti* at sites in northeastern Brazil (Molina *et al.*, 1996) and approximately half the native fish species of a natural lake in the Doce River valley in southeastern Brazil (Godinho *et al.*, 1994), where it was introduced together with the red piranha (*Pygocentrus nattereri*).

Kullander and Ferreira (2006) revised the taxonomy of the genus based on morphological characteristics, thereby identifying nine new species. These include the two from the Tocantins basin, namely *C. kelberi* and *C. piquiti*, which these authors also considered to be the very species introduced into the river basins of Minas Gerais state.

*Cichla* has frequently been introduced into river basins outside their native range, both in Brazil and other countries, whereupon they have often become invasive, being implicated in the local extinction of at least nine fish species (Zaret and Paine, 1973). Little is known, however, on the origins of these populations, their taxonomic identity or the number of individuals introduced in the first place. While *Cichla* were apparently introduced into northeastern Brazil in the late 1940s by government agencies for the purpose of installing fish breeding farms (Fontenele, 1948; Fontenele and Peixoto, 1979), no information is available as regards their river or rivers of origin. Subsequently, the fish also became well established in southeastern Brazil (Alves *et al.*, 2007), although it is not known whether these populations were established by animals that escaped from aquaculture farms or were deliberately released. As *Cichla* is a highly prolific fish, which adapts well to lentic conditions, its populations have expanded rapidly in hydroelectric reservoirs, floodplains and lagoons (Godinho *et al.*, 1994; Pompeu and Godinho 2003).

Invasive populations of *Cichla* were first registered in southeastern Brazil during the 1980s (Agostinho *et al.*, 1994), and since then, several impacts on indigenous fish populations have been reported (Pompeu and Godinho 2003). In the hydroelectric reservoirs of Furnas and Marimbondo, in the upper Paraná basin, the tucunaré, together with a second Amazonian species, the croaker (*Plagioscion squamosissimus*), have become the predominant members of the fish community (Santos *et al.*, 1994), and are already an important resource for local fisheries, being widely appreciated both for food and as a sport fish (Torloni, 1993).

Determining the invasion of introduced species is an important step in understanding and controlling the spread of invasive species (Chandler *et al.*, 2008). For example, by identifying the source of an invasive population, the most important transport vectors for introducing novel species into new areas, and which therefore need reinforced regulation, can be determined (Carlton, 2001). Specifying the invasion pathway can also lead to a deeper understanding of the criteria and mechanisms required for successful invasion (Vermeij, 1996).

The present study aims at specifying the genetic origin of the invasive tucunaré populations in four major Minas Gerais river basins by means of the molecular phylogenetic analysis of partial mtDNA regions (16s and Control Region - CR), besides providing additional molecular information on those native species not analyzed by Willis *et al.* (2007). The genetic data generated herein were compared with those from previous studies on *Cichla* from other South American sites (Oliveira *et al.*, 2006; Renno *et al.*, 2006; Willis *et al.*, 2007), in an attempt to elucidate the history of *Cichla* invasions.

## Material and Methods

###  Samples

**Figure 1:**
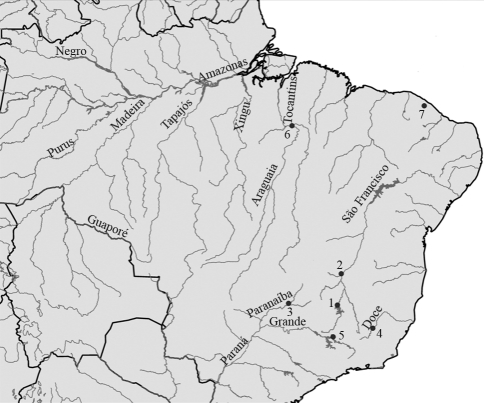
Map showing the collecting localities and Brazilian rivers in this study: (1) Morada Nova and Felixlândia in the Três Marias reservoir (São Francisco basin); (2) Marginal lake of the São Francisco (town of São Francisco, Minas Gerais); (3) Itumbiara reservoir, Paranaíba River (Minas Gerais); (4) Dom Elvécio Lake in the Doce River State Park (Minas Gerais); (5) Furnas reservoir, Grande River (Upper Paraná River Basin, Minas Gerais); (6) Tucuruí reservoir, Tocantins River (Pará); (7) Catu lake, Aquiraz (Ceará).

Tissue samples (fins) were collected from indigenous (Amazon basin, n = 41) and non-indigenous (non-Amazonian rivers, n = 103) specimens of tucunaré and preserved in 90% ethanol. Vouchers were preserved in a 30% formaldehyde solution and then transferred into an 80% ethanol solution. In the Amazon basin, fishes were collected from the Tucuruí reservoir (site 6 - Figure 1) on the Tocantins River - the eastern limit of the *Cichla* range - and from the Solimões River in the western Amazon basin. Non-indigenous specimens were collected from four different river systems in southeastern Brazil (Table 1, Figure 1) and one site in northeastern Brazil. DNA sequences from GenBank (Table 1) were included in the analyses, in order to compare our data with those available for other regions and species.

###  Taxonomic identification

All specimens were identified, according to Kullander and Ferreira (2006). Both *C. kelberi* and *C. piquiti* were collected from the Tucuruí and Itumbiara reservoirs (Figure 1: sites 3 and 6), whereas only *C. kelberi* was collected at sites 1 and 4, and *C. piquiti* was the only species found at sites 2 and 5. Specimens collected in Catu lake (site 7) could not be reliably identified based on Kullander and Ferreira (2006).

###  Genetic procedures and data analysis

Tissue samples were digested using proteinase K, and DNA isolated by phenol/chloroform purification (Sambrook *et al.*, 1989). Two mitochondrial markers were amplified, namely 16S rDNA (16S) and Control Region (CR), as both had previously been used in studies on population genetics of *Cichla* (Oliveira *et al.*, 2006; Willis *et al.*, 2007). A portion of 16S was amplified with the primers 16S-L1987 (5'-GCC TCG CCT GTT TAC CAA AAA C-3') and 16S-H2909 (5'-CCG GTC TGA ACT CAG ATC ACG T -3'; Palumbi *et al.*, 1991), and a portion of the CR was amplified with the primers L (5'-AGAGC GTCGGTCTTGTAAACC-3', Cronin *et al.*, 1993, and H16498 (5'-CCT GAA GTA GGA ACC AGA TG-3', Meyer *et al.*, 1990). These primers amplify PCR products that are approximately 500 bp (16S) and 460 bp (CR) in length. After the PCR reaction (16S: 94 °C for 3 min, 30 cycles at 94 °C for 1 min, 55 °C for 1 min and 72 °C for 3 min, followed by an extension of 72 °C for 7 min; CR: 94 °C for 4 min, 50 °C for 30 s, 72 °C for 2 min, 30 cycles at 94 °C for 15 s, 56 °C for 30 s and 72 °C for 2 min, with an extension of 72 °C for 10 min), 1-2 μL of the PCR product were used in the DNA sequence reaction, according to manufacturer's instructions (Big Dye Terminator Mix). The reactions were analyzed in an automated DNA sequencer (ABI 377 or ABI 310, Perkin Elmer) and the DNA sequenced in both directions. The nucleotide sequences generated in this paper were deposited in GenBank under the accession numbers DQ779579-DQ779586, FJ904286- FJ904291 for 16S rDNA and Control Region FJ890798- FJ890816.

The chromatograms were checked by manual inspection and aligned using default parameters in the ClustalW software included in the Bioedit program (Hall, 1999).

Phylogenetic trees were generated in a Bayesian framework MrBayes (Huelsenbeck *et al.*, 2001, 2002). Confidence for each node was verified by posterior probabilities for MrBayes. A PAUP 4* version 4.0b10 for Unix was also used for parsimony analysis (Swofford, 2003). The 16S and CR data sets were analyzed separately in order to incorporate as much information as possible from previous studies. Prior to phylogenetic analyses, selection of the nucleotide substitution model was done based on the Akaike information criterion (Akaike, 1973) using the perl script - mraic.pl - version 1.4.3 (Mraic.pl. program distributed by Nylander JAA, Uppsala University) in conjuntion with PHYML (Guindon and Gascuel, 2003). The evolutionary models used for rRNA 16S and Control Region were HKY (Hasegawa *et al.*, 1985) and GTR (Rodriguez *et al.*, 1990), respectively, both combined with the assumption that a proportion of sites are invariable (I), and that rate variation is modeled by gamma distribution (Yang, 1994). The posterior distribution of trees was approximated by using the Markov chain Monte Carlo as implemented in MrBayes. We ran four independent runs with four parallel chains, where each run started from a randomly chosen tree with 4 million generations each, and was sampled every 1000th generation. The first 1 million generations were discarded in each run as being a burn-in stage. Trees and parameter values from the four runs were pooled and a majority-rule consensus tree was calculated.

The major haplotypes identified by Oliveira *et al.* (2006), Renno *et al.* (2006) and Willis *et al.* (2007), were used to determine the genetic origin and species of each introduced population (non-indigenous), as well as the taxonomic distinctness of the Tocantins population, as suggested by Kullander and Ferreira (2006). *Geophagus brasiliensis* was used as outgroup in both analyses.

## Results

After trimming ambiguous ends, sequences of 410 bp and 359 bp were obtained, respectively, for 16S and the Control Region. The CR was more variable than 16S, with 157 variable sites, of which 102 were informative for parsimony analysis, as opposed to 50 variable sites with 17 informative for 16S. As topology and branch confidences in maximum parsimony were very similar to Bayesian analysis, only the later is shown in the present work.

Despite fewer specimens analyzed in relation to the Control Region, the Bayesian cladograms of the two markers were, in general, congruent.

###  16S analysis

The indigenous species from the Tocantins River formed two distinct clusters, *C. piquiti*, and *C. kelberi* significantly supported by Bayesian posterior probabilities (PP) of 0,95 and 0,99, respectively. The tucunarés from a marginal lake of the São Francisco and the Itumbiara and Furnas reservoirs (Figure 1; sites: 2, 3 and 5) are all included within the *C. piquiti* clade.

**Figure 2:**
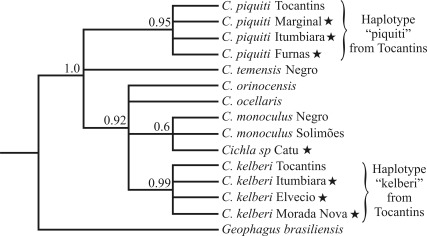
Phylogenetic tree based on 16S sequences in *Cichla*, and on haplotypes from the Tocantins River and several sites in southeastern Brazil, as well as Catu lake in northeastern Brazil. Selected haplotypes from the Amazon drainage (GenBank - see Table 1) were also used for comparison. Numbers above branches represent Bayesian posterior probabilities. Stars means non-indigenous specimens.

On the other hand, the data also indicated that tucunarés from the Itumbiara, Elvécio and Três Marias reservoirs (Figure 1: sites: 1, 3 and 4) were highly associated (PP = 0,92) to *C. kelberi* from the Tocantins River. In addition, the *C. kelberi* group was strongly connected to non-indigenous *Cichla* sp. from Catu lake, as well as to other unresolved lineages, such as *C. monoculus* from the Negro and Solimoes Rivers, *C. ocellaris* and *C. orinocensis* (PP = 0.92). Conversely, *C. temensis* from the Negro River was not associated to any other group, appearing as an independent indigenous lineage (Figure 2).

###  Control region analysis

**Figure 3:**
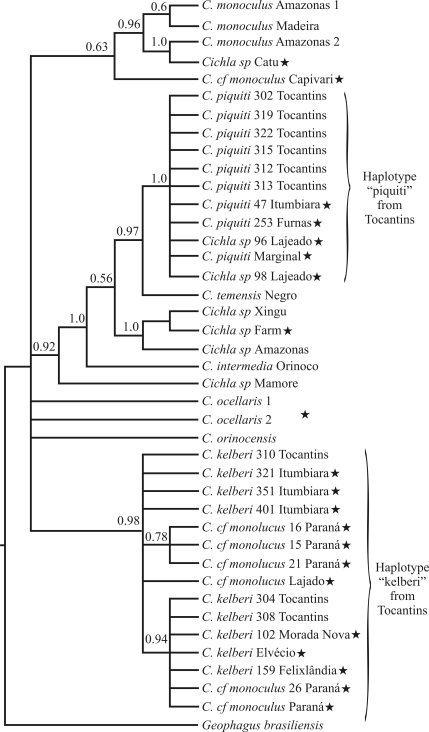
Phylogenetic tree based on control region (CR) sequences in *Cichla* spp, and on haplotypes from the Tocantins River and several sites in southeastern Brazil, as well as Catu lake in northeastern Brazil. Selected haplotypes from the Amazon drainage (GenBank - see Table 1) were also used for comparison. Numbers above branches represent Bayesian posterior probabilities. Stars means non-indigenous specimens.

In the control region analysis, the indigenous species from the Tocantins River also proved to be separated into two distinct clusters, *C. piquiti* and *C. kelberi,* supported by high posterior probabilities of 1.0 and 0.98, respectively*.* Furthermore, specimens from the *C. piquiti* clade were grouped together with tucunarés from the Itumbiara, Furnas, Marginal and Lajeado reservoirs (PP = 1.0). Interestingly, sustained by high posterior probability (PP = 0.97), *C. temensis* from the Negro River, which was unresolved by 16S analysis, appeared as a sister group of the *C. piquiti* group. Control Region data also suggested a close relationship among *C. intermedia* from the Orinoco, *Cichla* sp. from the Mamoré, and a group formed by *Cichla* sp from the Amazonas and Xingu (two unidentified native specimens), as well as the *Cichla* sp. farm species. This latter species (*Cichla* sp Farm), which is firmly connected (PP = 1.0) to native tucunarés from the Xingu and Amazonas, was denominated *C. temensis* by Oliveira *et al.* (2006). The unidentified native specimen from the Mamoré River is a basal specimen in this group. *Cichla ocellaris* and *C. orinocensis* appeared as independent unresolved lineages. On the other hand the *C. kelberi* specimens were all strongly grouped (PP = 0.98) with several non-indigenous tucunarés, such as those from Itumbiara, Paraná, Três Marias (Morada Nova and Felixlândia) and the Dom Elvécio lake. The data also showed a very well supported subgroup (PP = 0.94), this including a non-indigenous specimen of *C. kelberi* from the Tocantins, grouped together with introduced specimens from Três Marias (Felixlândia and Morada Nova; Figure 1: site 1), the Doce River (Dom Elvecio lake) (Figure 1: site 4) and Itumbiara (Figure 1: site 3). Only one haplotype was detected in all the introduction-sites, with the exception of Itumbiara, with 3 distinct CR haplotypes for *C. kelberi* (Figure 3).

The unidentified specimens from Ceará (Catu Lake) clustered with *C. monoculus* haplotypes from the Madeira and Amazonas Rivers, with high posterior probabilities (Figure 3: PP = 0.96).

## Discussion

The phylogenetic results of this study confirm that the introduced populations in the river basins of southeastern Brazil (Minas Gerais) all originated from the Tocantins River. In addition, we confirmed the genetic distinctness of the two *Cichla* species from the Tocantins (*C*. *piquiti* and *C. kelberi*), from those of the Amazon basin.

Fontenele (1948) and Fontenele and Peixoto (1979) reported that government agencies had translocated tucunarés to northeastern Brazil in order to improve the frail fish fauna in this region, this process then being repeated in southeastern Brazil. Our genetic data contradict this statement, given that the haplotypes of the introduced population from Ceará (Catu lake; northeastern Brazil) clustered with samples from the Amazon and Madeira Rivers, thus indicating the taxonomic identification of this species as *C. monoculus*, and the occurrence of at least two independent translocations. Additional samples from Ceará and other northeastern sites are required to confirm this difference.

The fact that the Itumbiara reservoir was the only locality in Minas Gerais where two species of *Cichla* occurred in sympatry (Table 1), as well as the only site where three different CR haplotypes for *C. kelberi* were detected (Figure 3), suggests that this was the target of more intensive translocations, and thus may represent the place of origin of the stocks of other localities in Minas Gerais.

Unexpectedly, different species, *C. kelberi* and *C. piquiti*, were recorded at sites 1 and 2, respectively, despite being located in the same river basin (São Francisco), thereby suggesting that *Cichla* is not widely dispersed in this region, possibly through initially introducing only a few specimens at each site, which would also account for only one species being recorded in most sites in Minas Gerais.

The success of introduced species has been generally attributed to their capacity of adapting to novel environments (Stockwell *et al.*, 2003; Alcaraz *et al.*, 2005). For example, the invasive freshwater fish *Lepomis gibbosus* has managed to adapt to different habitat-zones in reservoirs (Bhagat *et al.*, 2006). Likewise, its accentuated environmental plasticity and aggressive behavior may have enabled *Cichla* to successfully colonize new habitats throughout Brazil, in particular where habitats have been modified by dams. For instance, following the extinction of small native teleosts, the diet of tucunarés in Dom Elvécio Lake (site 4) shifted primarily to freshwater prawns (personal observation by the authors). Reflecting this, local anglers now prefer to use prawns as bait, rather than the usual techniques used elsewhere. Extirpation of the introduced stocks of tucunaré is probably impossible in most cases, but understanding the dynamics of these invasions might be helpful in developing management strategies for these and other non-indigenous species.

With the exception of a review by Kullander and Ferreira (2006), the introduced *Cichla* in Minas Gerais are still being misidentified as *C. temensis*, *C. ocellaris* and *C. monoculus* (*e.g.*, Alves *et al.*, 2007). None of these species were, however, detected at any of the four sites from Minas Gerais analyzed here. *C. temensis* from the Negro river seems to be a sister species of tucunaré from the Tocantins river. However, the Tocantins population represents an isolated stock, which reinforces the finding that this river basin is inhabited by two of the new species (*C. piquiti* and *C. kelberi*), as proposed by Kullander and Ferreira (2006).

In the present study, the analysis of two mitochondrial markers revealed that the two *Cichla* species endemic to the Tocantins River (Kullander and Ferreira, 2006) have been translocated extensively to sites in southeastern Brazil. Our study also provides additional phylogenetic information, since Willis *et al.* (2007) did not analyze *C. piquiti* or *C. kelberi*, and also elucidates certain aspects of the introduction of this highly invasive species.

## Figures and Tables

**Table 1:** Summary of species and sample sites analyzed, including information on whether fish were indigenous (I) or non-indigenous (N) in a particular region, and the number of 16S rDNA (16S) and control region (CR) haplotypes identified at each locality. References are given for previously published haplotypes that were obtained from GenBank. Abbreviations of the Brazilian states are: Amazonas (AM), Ceará (CE), Paraná (PR), Mato Grosso (MT), Minas Gerais (MG), Pará (PA), Tocantins (TO). *Numbers between brackets mean the total sample analyzed for each site; **Named by Oliveira *et al.* (2006) as *C. temensis.*

Species	Code	Location / State	I/N	16S/CR haplotypes*	Reference - GenBank
*C. kelberi*	Tocantins	Tucuruí reservoir, Tocantins River (PA)	I	1/3 (20)	This work
*C. monoculus*	Amazonas1	Amazonas River (AM)	I	0/1	Oliveira *et al.*, 2006 (AY836748)
*C. monoculus*	Amazonas2	Amazonas River (AM)	I	0/1	Willis *et al.*, 2007 (DQ841899)
*C. monoculus*	Madeira	Madeira River (AM)	I	0/1	Renno *et al.*, 2006 (DQ778669)
*C. monoculus*	Solimões	Solimões River (AM)	I	1/0 (1)	This work
*C. monoculus*	Negro	Negro River (AM)	I	1/0	Farias *et al.*, 1999 (AF049017)
*C. piquiti*	Tocantins	Tucuruí reservoir, Tocantins River (PA)	I	1/6 (20)	This work
*C. temensis*	Negro	Negro River (AM)	I	1/1	Farias *et al.*, 1999 (AF049019) Willis *et al.*, 2007 (DQ841929)
*C. ocellaris*	-	?	I	1/2	Sparks 2004 (AY263831) Renno *et al.*, 2006 (DQ778665) Willis *et al.*, 2007 (DQ841871)
*C. orinocensis*	-	?	I	1/1	Willis *et al.*, 2007 (DQ841866) Farias *et al.*, 1999 (AF049018)
*C. intermedia*	Orinoco	Orinoco River, Venezuela	I	0/1	Willis *et al.*, 2007 (DQ841833)
*Cichla* sp*.*	Mamore	Mamoré River, Amazon basin, Bolivia	I	0/1	Willis *et al.*, 2007 (DQ841908)
*Cichla* sp*.*	Xingu	Xingú River (PA)	I	0/1	Willis *et al.*, 2007 (DQ841946)
*Cichla* sp*.*	Amazonas	Amazonas River (AM)	I	0/1	Willis *et al.*, 2007 (DQ841937)
*Cichla* sp*.*	Lajeado	Lajeado reservoir Tocantins River (TO)	I	0/2	Oliveira *et al.*, 2006 (AY836743-44)
*C. kelberi*	Itumbiara	Itumbiara reservoir Paranaíba River (MG)	N	1/3 (18)	This work
*C. kelberi*	Morada Nova	Três Marias reservoir, Morada Nova district, São Francisco River (MG)	N	1/1 (12)	This work
*C. kelberi*	Elvécio	Dom Elvécio Lake, Doce River (MG)	N	1/1 (10)	This work
*C. kelberi*	Felixlândia	Três Marias reservoir, Felixlândia district, São Francisco River (MG)	N	0/1 (10)	This work
*C. piquiti*	Itumbiara	Itumbiara reservoir, Paranaíba River (MG)	N	1/1 (19)	This work
*C. piquiti*	Furnas	Furnas reservoir, Grande River (MG)	N	1/1 (17)	This work
*C. cf. monoculus*	Paraná	Paraná River (PR)	N	0/4	Oliveira *et al.*, 2006 (AY836717-20)
*C.* cf. *monoculus*	Capivari	Capivari reservoir, Paranapanema River (PR)	N	0/1	Oliveira *et al.*, 2006 (AY836730)
*C.* cf. *monoculus*	Itaipú	Itaipú reservoir, Paraná River (PR)	N	0/1	Oliveira *et al.*, 2006 (AY836739)
*C.* cf. *monoculus*	Lajeado	Lajeado reservoir, Tocantins River (TO)	N	0/1	Oliveira *et al.*, 2006 (AY836746)
*C. piquiti*	Marginal	Marginal lake, São Francisco River (MG)	N	1/1 (12)	This work
*Cichla* sp.**	Farm	Fish farm (MT)	N	0/1	Oliveira *et al.*, 2006 (AY836740)
*Cichla* sp.	Catu	Catu lake (CE)	N	1/1 (5)	This work
